# A Sagittal Patellar Angle Linear Equation Reflecting Patellofemoral Kinematics: Evaluation of Patellar Height at any Degree of Knee Flexion Angle

**DOI:** 10.1111/os.13166

**Published:** 2021-11-16

**Authors:** Lu‐kuan Cui, Kai Kang, Xiao‐zuo Zheng, Shi‐gang Jiang, Wen‐tao Huang, Shi‐jun Gao

**Affiliations:** ^1^ Department of Orthopedics Third Hospital of Hebei Medical University Shijiazhuang China; ^2^ First Department of Traumatic Orthopedics Cangzhou Hospital of Integrated Traditional and Western Medicine·Hebei Province Cangzhou China; ^3^ Key Laboratory of Biomechanics of Hebei Province Shijiazhuang China

**Keywords:** Knee flexion angle, Linear equation, Patellar height, Sagittal patellar angle

## Abstract

**Objective:**

To confirm whether a novel sagittal patellar angle linear equation used for evaluating patellar height by calculating expected sagittal patellar angle (SPA) at any degree of knee flexion angle is suitable for patients older than 17 years and its reliability compared with other commonly used methods.

**Methods:**

From September 2016 to September 2019, a total number of 202 consecutive outpatients' knee lateral X‐ray radiographs were retrospectively measured and evaluated using a recently proposed linear equation *Y = 1.94 + 0.74* × *knee flexion* *(KF) angle*. Patients were divided by ages into ayounger group, whose ages were between 17–49 years, and an older group, whose ages were older than 49 years, which has not been validated in the original study. Parameters such as KF, SPA, patella and patella tendon length and so on were measured on computer with picture archiving and communication system by two independent observers at an interval of 1 month. Insall‐Salvati (IS) index, Caton‐Deschamps (CD) index and Y value, correlation coefficients were calculated and compared using SPSS 22.0 software.

**Results:**

In the younger group, 143 patients (165 knees) were included, ages were 17–49 (31.62 ± 11.38) years, males/females were 70 (48.95%)/73 (51.05%), left knees/right knees were 83 (50.30%)/82 (49.70%), mean value of Y was 31.50° ± 10.07°, and SPA was 34.38° ± 12.38°, mean value of IS was 1.06 ± 0.17, mean value of CD was 1.04 ± 0.18. While in older group, 59 patients (78 knees) were included, ages were 50–60 (mean 54.61 ± 2.99) years, there were 32 males (54.24%) and 27 females (45.76%), 42 knees were left (53.85%) and 36 knees were right (46.15%), mean values of Y and SPA were 25.90° ± 11.55° and 29.36° ± 14.22°, mean IS index in older group was 1.06 ± 0.18, mean CD index was 1.00 ± 0.16. Intra‐ and inter‐observer reliabilities of Y in younger and older groups were 0.999, 0.999, 1.000 and 0.999, meaning high reliability and reproducibility, but low Pearson's correlation coefficients with IS and CD index were showed as −0.213 and − 0.216 in younger group and − 0.113 and − 0.316 in older group.

**Conclusions:**

In patients older than 17 years, the linear equation *Y = 1.94 + 0.74 × KF* is a reliable and practical method to evaluate SPA regardless of age and knee flexion angle, but has weak correlation coefficients with the IS and CD index.

## Graphical abstract

To confirm the effectiveness of a novel sagittal patellar angle equation used for evaluating patellar height by calculating expected sagittal patellar angle(SPA) with knee flexion angle.

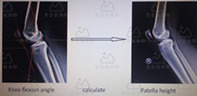

## Introduction

The position of patella contributes greatly to knee stability, and patellar height plays an important role in surgery design for total knee arthroplasty (TKA), high tibial osteotomy (HTO) and other operations around the knee joint. The correlations between patella alta and recurrent patella dislocation, osteoarthritis, and so on have been revealed. Patella baja (PB) would increase pressure of the patella, followed by negative effects like decrease of knee joint motion range, knee extensor weakness, chondromalacia and patellofemoral arthrosis^1^. The force between femoral trochlea and patella increases as the position of patella decreases. True PB is associated with bad clinical outcomes, but consequences of pseudo PB may be not obviously different from the normal patella and needs no further surgical treatment^2^. Furthermore, the relationship between patellar height and knee instability has been recognized by researchers. Therefore, precise measurement of patellar height is important for clinicians to evaluate patella instability and make plans for surgical procedure.

Based on the evaluation of radiographs proceeded at 30° knee flexion, Blumensaat proposed one of the first patellar height evaluation methods. After that, many kinds of methods, such as Insall–Salvati (IS) index, Blackburne–Peel (BP) index, Caton–Deschamps (CD) index and their modification ratios, have been proposed to assess patellar height, the reliability and reproducibility have also been compared, but until now, no consensus has been reached on which method is a gold standard for patellar height assessment^3^, ^4^. In a recent comparative study, Verhulst *et al*.^5^ examined the most popularly used methods for measuring patellar height on different fluoroscopic images and demonstrated that the IS index displays better than other methods. In consideration of that, the CD index has an advantage in measuring patellar height regardless of tibial tubercle position, knee flexion angle, knee joint size and image enlargement and quality and could be performed better than BP^6^, while other methods were not used as commonly as these two indexes or used especially for certain conditions such as a modified CD ratio, was shown to be useful for evaluating patellar height after TKA^6^. We only took the IS index and CD index in our study.

Most of the methods commonly used need to measure patellar height on standard knee flexion lateral radiographs at a certain degree which sometimes is hard to be exactly performed in clinical work, therefore, making it difficult to compare pre‐ and postoperative effects or different surgical techniques. To resolve this problem, a patellar height measurement method that could be carried out at any knee flexion angle and then the height of patella could be calculated for a certain flexion degree is needed. Dan *et al*.^7^ made use of 44 healthy volunteers without previous operations and knee disorders whose ages were within the range of 17–34 years to define new patellar height measurement methods including three linear and three polynomial equations, the authors also demonstrated the equations were eligible for patella instability patients and useful for reflecting patellofemoral kinematics. However, to our best knowledge, whether one of these six equations testified by the proposers to be more reliable than others, that is *Y (expected sagittal patellar angle) = 1.94 + 0.74 × knee flexion (KF) angle*, could be applied for all patients older than 17 years, especially older than 49 years has not been studied in the literature. Thus, the purposes of this research were to: (i) corroborate the validity of the linear equation for calculating sagittal patellar angle (SPA) in patients; (ii) confirm whether this linear equation is suitable for patients with different ages; and (iii) compare effectiveness of the new method with the IS index and CD index. The hypothesis before the study was that the established equation would be useful for almost all patients older than 17 years regardless of age, but a slight difference may exist among different methods.

## Materials and Methods

### Inclusion and Exclusion Criteria

Outpatients who had been examined using knee lateral X‐ray from September 2016 to September 2019 were retrospectively screened in our picture archiving and communication system (PACS, Neusoft, Shenyang, China). The inclusion criteria were as follows: (i) patients older than 17 years; (ii) patients who had not undergone any operation around the knee; and (iii) patients with a true lateral X‐ray image of which the two posterior femoral condyles were presented as superimposed, showing an appearance of one posterior condyle of the femur^8^. We excluded radiographs with advanced knee degeneration changes, which may impact measurement or fracture and injury around knee joint. Radiographs with knee rotation were also rejected as this would result in measurement error.

Patients were divided into two groups by ages: the younger group, whose ages were between 17–49 years^7^, and the older group, whose ages were older than 49 years. KF angle between 10° and 80°^6^ was included, as the CD index could be used, and this was within the scope of 7.05°–113.18° which were found as lower and upper limits of this linear equation would be testified^7^.

### Parameters Definition and Measurement

#### Knee Flexion (KF) Angle

**Fig. 1:**
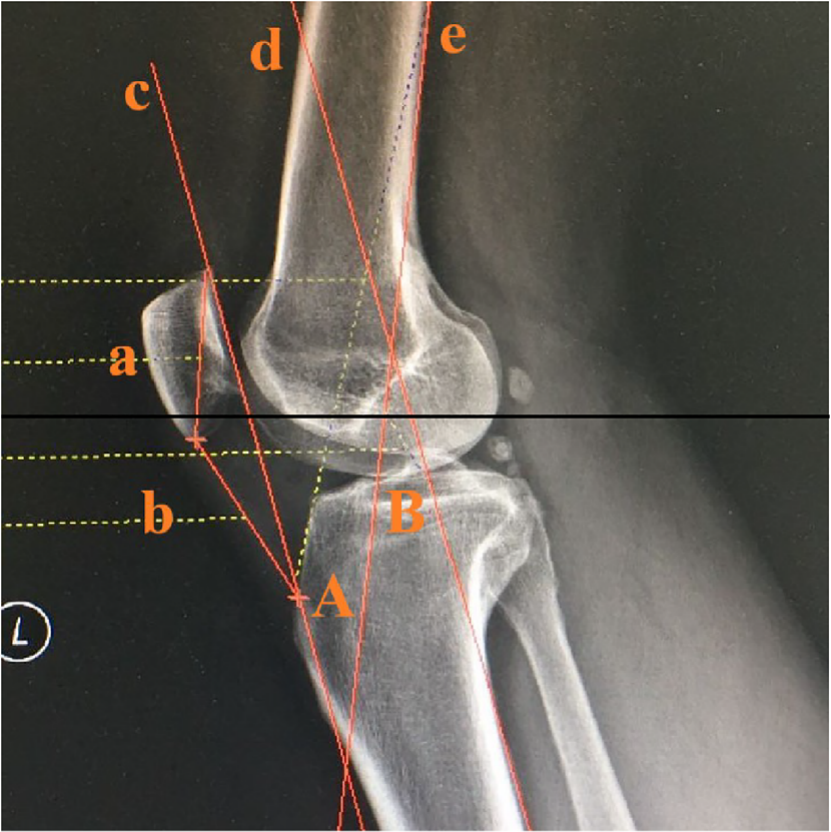
Insall–Salvati (IS) index, Sagittal Patellar Angle (SPA) and Knee Flexion (KF) Angle: (a) the length of patella. (b) the length of patella tendon. IS index was defined as (b)/(a). (c) tangent line of the articular surface of patella. (d) tangent line of tibia post cortex. (e) tangent line of femur post cortex. (A) named as SPA, the subtended angle of (c) and (e). (B) KF angle which was formed by (d) and (e).

We defined KF as an angle formed by two lines which was tangent with the post cortex of femur and tibia respectively similar with the method of Dan *et al*.^7^. It was recorded by degree (°) to reflect the angle of knee flexion on lateral X‐ray radiographs (Fig. 1).

#### Sagittal Patellar Angle (SPA)

SPA was defined as an angle between the tangent line of the femur post cortex and the tangent line of the articular surface of the patella as described above^7^. This subtended angle of the two lines reflects patellar height indirectly (Fig. 1).

#### Insall–Salvati (IS) Index and Caton–Deschamps (CD) Index

**Fig. 2:**
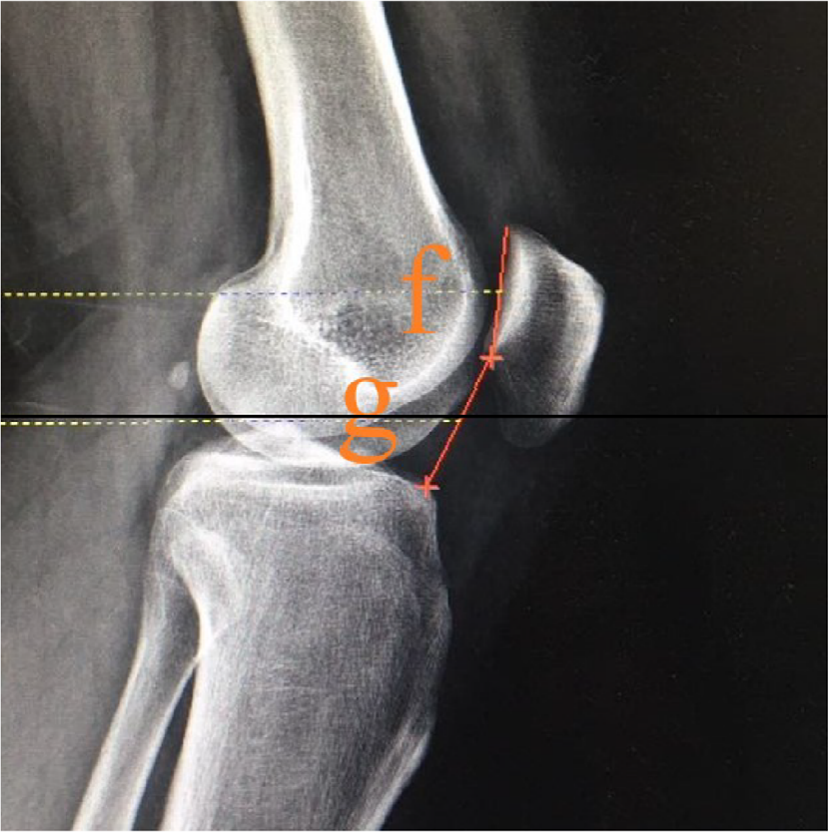
Caton–Deschamps (CD) index: (f) the length of articular surfice of patella. (g) the length from lower pole of articular surface of the patella to tibial plateau anterosuperior angle. CD index was defined as a ratio that (g) divided by (f).

These two indexes are commonly used methods in clinical practice for evaluating patellar height. The IS index is defined as a ratio of patella tendon length and patella length (Fig. 1). CD index is a ratio that the length from the lower pole of articular surface of the patella to the tibial plateau anterosuperior angle divided by articular surface length of the patella (Fig. 2). The length of each subject was recorded in millimeters (mm).

All parameters were measured by two observers twice at an interval of 1 month using PACS automatically within two decimals of the length value. *Y* was calculated by the linear equation *Y = 1.94 + 0.74 × KF*.

### Statistical Analysis

SPSS 22.0 (IBM, Armonk, NY, USA) was used to analyze the parameters. Values were showed by mean ± standard deviation (SD). Correlations between KF, SPA, Y and inter‐, intra‐observer were evaluated by Pearson's correlation coefficient. Correlations between different methods among Y, IS index and CD index were also evaluated using Pearson's correlation coefficient. Weak, moderate, strong and excellent correlation coefficients were divided as ≤0.5, 0.5–0.7, 0.7–0.9 and ≥0.9 respectively. A *P* value of 0.05 was considered as a representation of significant difference.

## Results

### General Results

According to the inclusion and exclusion criteria, a total number of 202 patients (243 knees) were included in this study. In the younger group, 143 patients (165 knees) were included, ages were within the range of 17–49 (31.62 ± 11.38) years, males/females were 70 (48.95%)/73 (51.05%), left knees/right knees were 83 (50.30%)/82 (49.70%). In the older group, 59 patients (78 knees) were included, ages were from 50–60 (mean 54.61 ± 2.99) years, there were 32 males (54.24%) and 27 females (45.76%), 42 knees were left (53.85%) and 36 knees were right (46.15%) (Table 1).

**TABLE 1:** General results of the younger and older groups

Groups	Patients (knees)	Age (years)	Males/Females	Left/Right
Younger	143 (165)	31.62 ± 11.38	70 (48.95%)/73 (51.05%)	83 (50.30%)/82 (49.70%)
Older	59 (78)	54.61 ± 2.99	32 (54.24%)/27 (45.76%)	42 (53.85%)/36 (46.15%)

### Intra‐ and Inter‐observer Reliability of the Liner Equation

Both the intra‐ and inter‐observer reliabilities of the liner equation in the younger and older groups are shown in Table 2. From the Table, we could find that the intra‐ and inter‐observer reliabilities of observer 1 and 2 in the younger group were also 0.999. In the older group, the intra‐observer reliabilities of Y values of observer 1 and 2 were 1.000 and 0.999, while inter‐observer reliability was 0.999.

**TABLE 2:** Measurement results and the intra‐ and inter observer reliabilities of the two groups

Groups	Intra‐observer reliability		Inter‐observer reliability	Y (°)	SPA (°)	IS	CD
	Observer 1	Observer 2					
Younger	0.999	0.999	0.999	31.50° ± 10.07°	34.38° ± 12.38°	1.06 ± 0.17	1.04 ± 0.18
Older	1.000	0.999	0.999	25.90° ± 11.55°	29.36° ± 14.22°	1.06 ± 0.18	1.00 ± 0.16

Y represents the value calculated by the linear equation *Y = 1.94 + 0.74 × knee flexion* angle.

CD, Caton–Deschamps index; IS, Insall–Salvati index; SPA, sagittal patellar angle; Y, SPA, IS and CD, mean value of the four measurement data performed by two observers twice respectively.

### Outcomes of Measurement and Calculation Values

The mean value of Y was 31.50° ± 10.07° and SPA was 34.38° ± 12.38° in the younger group. The mean value of IS was 1.06 ± 0.17, and that of CD was 1.04 ± 0.18 in this group. While in the older group, mean values of Y and SPA were 25.90° ± 11.55° and 29.36° ± 14.22°. Mean IS index in older group was 1.06 ± 0.18, while mean CD index was 1.00 ± 0.16 (Table 2).

### Correlation Analysis Results

**Fig. 3:**
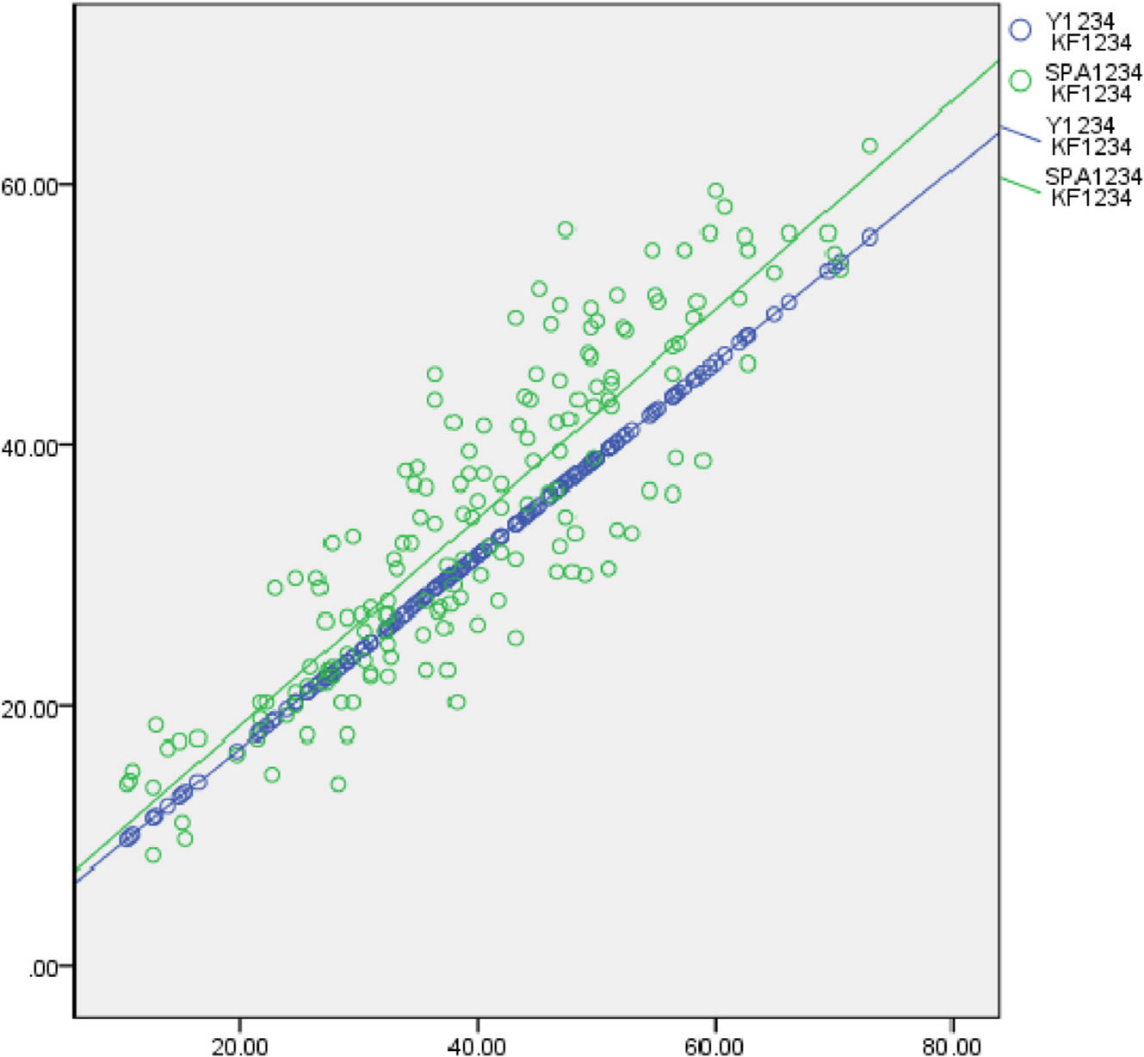
Correlations of mean Y and SPA values with KF values in younger group. Since all parameters were measured by two observers for two times at an interval of 1 month, Y_1234_, KF_1234_ and SPA_1234_ represent the mean values of the four times measurement results of the two surveyors respectively in the process of calculation with SPSS software. It can be seen from this figure that Y has a good consistency with SPA in younger group regardless of knee flexion angle.

**Fig. 4:**
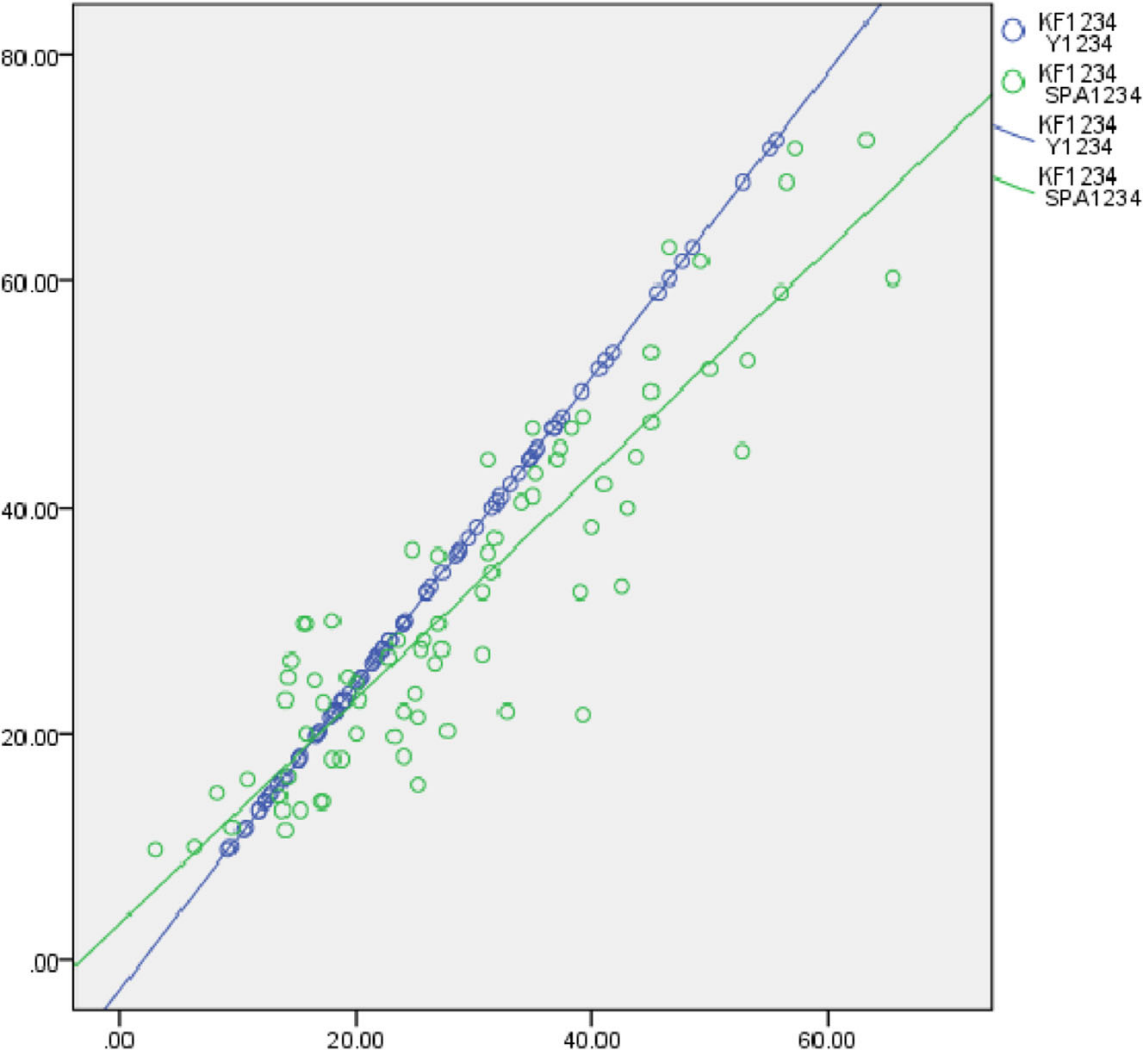
Correlations of mean Y and SPA with KF in older group. Y_1234_, KF_1234_ and SPA_1234_ represent the mean values of the four measurement data performed by two observers twice at an interval of 1 month respectively. It can be seen from this figure that in the older group, no matter how the knee flexion angle was, Y and SPA had a good consistency.

In the group whose ages were younger than 49 years, the correlation coefficient between Y and SPA was 0.879 (*P* < 0.01), meaning that Y value has a strong correlation with SPA (Fig. 3). Correlation coefficient between these two values was 0.903 (*P* < 0.01) in the older group, a strong correlation was shown (Fig. 4). However, correlation coefficients between Y/IS and Y/CD were −0.213 and −0.216 among younger patients respectively, meaning that Y has negative correlations with the IS and CD indexes, but the correlations were weak (Table 3). On the other hand, these two correlation coefficients in the group older than 49 years were − 0.113 and − 0.316 using Pearson's correlation coefficient, meaning a weak negative correlation (Table 4).

**TABLE 3:** Pearson's correlation coefficients of Y with other methods in younger group

	Y/SPA	Y/IS	Y/CD
*r*	0.879	−0.213	−0.216
*P*	0.000	0.006	0.005

*r*: Pearson's correlation coefficient. *P* < 0.05 was considered as significant difference.

**TABLE 4:** Pearson's correlation coefficients of Y with other methods in older group

	Y/SPA	Y/IS	Y/CD
*r*	0.903	−0.113	−0.316
*p*	0.000	0.323	0.005

*r*: Pearson's correlation coefficient. *P* < 0.05 was considered as significant difference.

## Discussion

### Reasons for Including IS and CD Indexes in Comparison

Disorders of patella position correlated with patellar height would cause chondromalacia, knee pain or subluxation subsequently^9^, and may impact the reaction force between the patella and the femur condyle groove when the knee is in flexion, on account of the force increasing with the patella decreasing^6^, ^10^. Many patellar height assessment methods have been proposed and modified for common application, whereas limitations still exist and no one way could be used to evaluate all situations. Since the landmark used for the CD index (with a normal range of 0.6–1.2) could not be identified after TKA, Caton *et al*.^6^ modified the CD index to a ratio that the distance from the distal point of the patella to a new landmark T' marked as the line passing through the head of fibula but perpendicular to the tibial posterior cortex and intersecting with tibial anterior cortex to assess the patellar height after TKA.

To eliminate influence of factors such as patients' height and weight, calculation and radiographs magnification, another simpler method was discovered by Portner *et al*.^11^ called Plateau‐Patella Angle (PPA). It was defined as an angle formed by the line connecting the posterior end of medial tibial plateau and lower pole of the articular surface of patella, and a tangential line of the medial tibial plateau. This method was validated to be an eligible method for assessing patellar height after HTO, but a −0.67 correlation was found between PPA reduction and tibial slop increase, therefore, PPA may overestimate patellar height after HTO^12^. Ellington and his colleagues indicated that PPA has a higher reproducibility and advantage of simpler implementation compared with IS, BP and CD index^9^. However, plateau position would alter after HTO and may subsequently result in changes of patellar height measurement^13^.

Inconsistency of X‐ray radiograph, computerized tomography or magnetic resonance imaging used in measurement, especially accurate slice used to measure bone and cartilage for patients with patella instability, and different experience among observers may lead to conflicting conclusions. Smith *et al*.^14^ compared reliabilities among different indexes and discovered a better superiority of CD index than others. However, this was controversial with Verhulst *et al*.^5^ in whose opinion IS was the most reliable method among commonly used measurements for its higher reliability and reproducibility^15^. The relationship of another popularly used method to evaluate patellar height, that is patellotrochlear index, with other methods was also inconsistent among researchers^16^, ^17^, ^18^.

As mentioned above, both the IS and CD indexes are ratios of which high inter‐observer agreements have been validated^19^. They are typical indexes for patellar height evaluation reflecting the length of the patella tendon or the distance between distal pole of patella and tibial plateau respectively and could be assessed without error derived from physical size and radiograph magnification. The IS index is also independent of knee flexion and could detect true PB but not pseudo PB caused by elevation of joint line after some surgical procedures. However, this disadvantage would be supplemented by the CD index. Furthermore, the IS and CD indexes will not be affected when they are used to evaluate patellar height after TKA with rotation of the femoral component. The combination of the IS and CD indexes would provide accurate assessment of patellar height^20^. Their simplicity and accuracy make them widely used in clinics. Therefore, it is these two simple and convenient indexes instead of others that we took into consideration in this study.

### Advantages of the Novel Linear Equation

Another problem of conventional indexes is the rigid flexion angle when patients are positioned to take fluorescence images, which is difficult to guarantee in clinical practice, but, if not, would result in inaccurate measurement outcomes. Quadriceps force may alter with different knee flexion angles and subsequently influence the measurement results. Laugharne *et al*.^21^ reported the difference under quadriceps in activation or not was negligible in patellar height measurement, however, this is still in dispute.

To overcome these restrictions, Dan *et al*.^7^ defined three polynomial and three linear equations to calculate patellar height on the sagittal plan. In their study, the authors described six equations by recruiting 44 normal volunteers and verified the equations with 31 patients who had undergone tibial tubercle osteotomy. Final results turned out to be that these novel measurements got sensitivity and specificity of 77% and 95% respectively with two residual SD to define a normal expected value range, could reflect patellofemoral kinematics at the same time. Most importantly, SPA, the one testified to be the easiest to use, the most reliable and had the least residual error among all six defined equations, may be superior, as clinicians could use it to calculate expected angle at any knee flexion angle even if the lateral radiograph was not taken under a standard position or at a specific knee flexion angle. As the examples given by the authors in their original literature, KF and SPA values can be measured with lateral knee radiograph. The corresponding Y could be calculated by the linear equation at a particular value of KF, and then compared with the actual SPA. When the measured SPA is one residual SD which was presented by the author as 3.59 higher than the Y value, it would be considered as patella alta. The percentage of patients with patella alta identified by this linear equation was 83.9% shown in their study. Our results also demonstrated the reliability of SPA and KF linear equation. However, our results were not absolutely consistent with that of Dan *et al*.^7^, one major interpretation may be that our study was a retrospective research, thereby we could not rigidly confine the position without rotation previously when patients had been taking X‐ray images. The results showed high reliability and reproducibility of this new method, but when compared with IS and CD methods, low negative correlations were shown.

### Significance of the Validation based on Age group

Although this novel patellar height measurement method reflecting patellofemoral kinematics has the advantages described above and has been validated in patients following tibial tubercle osteotomy, the authors recognized that the results may vary across ethnicities and did not verify whether the equation is applicable to patients over 49 years of age. The effectiveness of this method was also not compared with other commonly used indexes such as IS and CD. So we performed this research to confirm that. The research objects included were divided into two groups: the younger group with an age range from 17 to 49 years, which was consistent with the original literature, and the group older than 49 years. Statistical analysis was conducted on the younger group first, and then the group older than 49 years was further verified after its validity could be confirmed by younger patients. If validated, our results would complement the original research outcomes for clinical application. As expected, some important findings were obtained by our imaging measurement and comparative study: (i) the linear equation *Y = 1.94 + 0.74 × KF* has considerable reliability and reproducibility but weak negative correlations with IS and CD methods; and (ii) this method could be used for older patients, even if he or she was older than 49 years, which was not testified before, and under conditions regardless of knee flexion angle to calculate SPA.

### Limitations

One limitation of the present study is that we evaluated the data without identifying weight characteristics, and we acknowledged that may have some influence on the accuracy of the outcome. However, Laugharne *et al*.^21^ reported no significant difference with relaxed or flexed position. Second, we found the measured and calculated sagittal angles of the patella were not completely consistent both in the original literature and our study. One reason we acknowledge is that we measured the KF angle by two lines tangent to the outside of femur and tibial post cortex, while inside tangent lines of post cortex were used in Dan *et al*.^7^ article. However, these two methods should provide similar results theoretically. A knee angle formed by the two lines of femur and tibial axes respectively through two centers of circles in the femur and tibial shafts may increase KF angle measurement accuracy, but this would make our measurement results incomparable with the results of the original authors. What is more, a similar tangent line method used in our study has been reported in previous literature^22^, ^23^, thus this should be a credible approach. Another limitation is that although the reliability and reproducibility of this novel method in preoperative patients was validated by our study, the effectiveness after special surgery such as TKA or HTO in which postoperative outcomes have a correlation with patellar height change was not confirmed. That should be carried out in future research. Last but not the least, our sample size of this retrospective study was small, which may also be a contributor to the discrepancy of correlations between Y values and the IS and CD indexes.

### Conclusions

Though correlation coefficients between the Y and IS and CD indexes were low, we cannot deny that this method using linear equation *Y = 1.94 + 0.74 × KF* to evaluate SPA has considerable reliability and reproducibility. It provides a novel method reflecting patellofemoral kinematics for patellar height evaluation pointed out by its proposers and is easy for clinicians to apply regardless of age in patients older than 17 years and knee flexion angle. However, the comparison with other methods like PPA and the validation of its effect after a special surgery such as TKA or HTO which were not carried out in this study need further research.

#### Authorship Declaration

All authors declare that all authors listed meet the authorship criteria according to the latest guidelines of the International Committee of Medical Journal Editors, and all authors are in agreement with the manuscript, and have no conflict of interest.
